# The Pulmonary Complications of Paraneoplastic Autoimmune Vasculitis in a Patient With Myelodysplastic Syndrome

**DOI:** 10.7759/cureus.9282

**Published:** 2020-07-19

**Authors:** Pooja S Jagadish, Anna-Carson R Uhelski, Jordan Redfield, Nicholas Thomson, Osarenren Ogbeide

**Affiliations:** 1 Internal Medicine, The University of Tennessee Health Science Center, Memphis, USA; 2 Medicine, Johns Hopkins Hospital, Baltimore, USA; 3 Medicine, The University of Tennessee Health Science Center, Memphis, USA; 4 Hematology and Oncology, The University of Tennessee Health Science Center, Memphis, USA; 5 Internal Medicine/Hematology/Oncology, Veterans Affairs Hospital, Memphis, USA

**Keywords:** myelodysplastic syndrome, mds, vasculitis, paraneoplastic, alveolar hemorrhage

## Abstract

Paraneoplastic autoimmune phenomena may occur in up to 30% of patients with myelodysplastic syndrome (MDS). We present the case of a patient with MDS who developed diffuse alveolar hemorrhage due to paraneoplastic autoimmune vasculitis.

The patient was a 55-year-old male who had been referred for outpatient hematology/oncology evaluation by his primary care physician for incidentally discovered thrombocytopenia. When he presented to the clinic, he reported new-onset chills, weakness, and night sweats. He endorsed a 20-pound weight loss over two months as well as two weeks of fatigue, exertional dyspnea, and epistaxis. He was noted to be ill-appearing and had bilateral pitting edema to the knees. Vital signs revealed a temperature of 102.3 °F, oxygen saturation of 84% on room air, and tachycardia to the 90s. Labs showed hemoglobin of 5.7 g/dL, hematocrit of 17.2 g/dL, and platelet count of 27 kµL. He was admitted to the hospital for blood and platelet transfusions, empiric antibiotics, and further diagnostic studies. The peripheral blood smear showed 4% blasts and frequent dyspoietic granulocytes. Bone marrow biopsy (BMB) was performed to differentiate between acute leukemia and myelodysplasia. BMB revealed myelodysplasia with excess blasts and erythroid predominance.During hospitalization, the patient developed acute hypoxemic respiratory failure due to bronchoscopy-confirmed diffuse alveolar hemorrhage from thrombocytopenia. His platelet count was 12 kµL. High-dose corticosteroids (2 mg/kg prednisone) were initiated for suspected paraneoplastic autoimmune vasculitis, pending BMB results. The patient steadily improved, was extubated, and had reduced oxygen and transfusion requirements.High-dose steroids were stopped, and the patient was started on decitabine chemotherapy with the ultimate goal of bone marrow transplantation. On day five of decitabine, the patient developed acute hypoxic respiratory failure requiring intubation as well as hypotension requiring vasopressors. Given that recurrent diffuse alveolar hemorrhage was again suspected, high-dose steroids were resumed upon transfer to the ICU. He continued to decompensate and ultimately experienced ventricular tachycardia requiring three separate episodes of cardiopulmonary resuscitation. Per the family’s wishes, he was palliatively extubated, and he expired an hour later.

Diffuse alveolar hemorrhage is a rare but potentially deadly pulmonary complication of MDS, stemming from a paraneoplastic autoimmune vasculitis. Patients who initially present with atypical autoimmune phenomena should raise suspicion for an underlying MDS, the presence of which can guide the promptness, extent, and duration of immunosuppressive therapy. Failure to expeditiously treat these patients with corticosteroids can lead to serious complications and death.

## Introduction

The World Health Organization defines myelodysplastic syndrome (MDS) as “… a group of diverse clonal hematopoietic disorders characterized by ineffective hematopoiesis, manifested by morphologic dysplasia in hematopoietic cells and bone marrow failure, refractory cytopenias, and by risk of progression to acute myeloid leukemia” [1].

Paraneoplastic autoimmune syndrome refers to cancer-related symptoms and signs, the exact etiology of which is not clear. Some have speculated that cancer cells produce bioactive substances that stimulate an autoimmune response [2]. Others have postulated that it may be related to medications used to treat cancer.

Autoimmune vasculitis, one such paraneoplastic autoimmune syndrome, can present in various forms and can sometimes predate the cancer diagnosis, initially presenting to rheumatology. This can take the form of cutaneous leukocytoclastic vasculitis, granulomatosis with polyangiitis (GPA, formerly Wegener’s granulomatosis), immunoglobulin A (IgA) vasculitis (formerly Henoch-Schönlein purpura), eosinophilic granulomatosis with polyangiitis (formerly Churg-Strauss syndrome), microscopic polyangiitis, and polyarteritis nodosa (PAN). With regard to MDS and vasculitis, Fain et al. have described 60 patients with cancer-related vasculitis, of which 21 had MDS [3]. Nine patients had PAN, nine had leukocytoclastic vasculitis, one had GPA, and one had microscopic polyangiitis. The authors have also noted that vasculitides associated with MDS resulted in more frequent renal involvement (p = 0.02) and steroid dependence (p = 0.04); remission was less frequently achieved in MDS-mediated vasculitis compared to vasculitides associated with other malignancies (p = 0.04) [3]. Immune-mediated hematologic presentations, such as anemia and thrombocytopenia from an MDS-associated paraneoplastic syndrome, is uncommon [4]. This report discusses the case of a 55-year-old male patient who developed an autoimmune vasculitis secondary to myelodysplasia.

## Case presentation

The patient was a 55-year-old male with a history of hypertension, hyperlipidemia, gout, tobacco use, and C5 cervical fusion surgery after a motor vehicle accident. He presented to the hematology/oncology clinic with acute-onset chills, weakness, night sweats, and intermittent headaches as well as a 20-pound weight loss over the past two months. He reported two weeks of fatigue and exertional dyspnea and noted intermittent epistaxis. The patient was tachycardic to the 90s, but vital signs were otherwise normal. He was alert and oriented, diaphoretic, and ill-appearing with 2+ bilateral pitting edema to the knees. He was admitted to the hospital for observation and further work-up.

**Figure 1:**
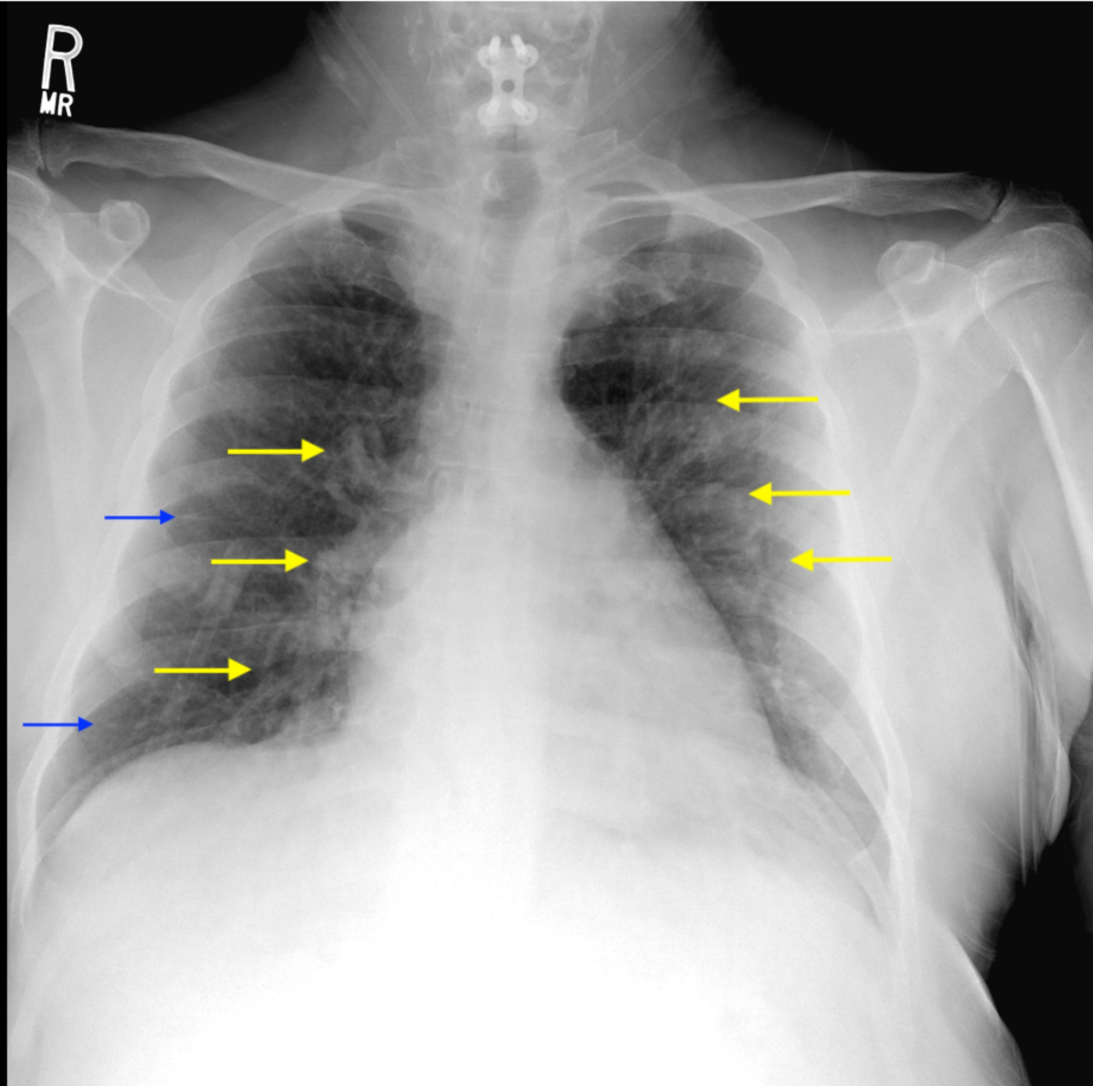
Chest X-ray of the patient The image displays the development of diffuse pulmonary interstitial edema/infiltrates, including perihilar vascular congestion (yellow arrows) and Kerley B lines (blue arrows)

Within a couple of hours of presentation, the patient’s temperature rose to 102.3 °F. He became increasingly tachycardic and desaturated to 84% on room air. Significant laboratory values can be seen in Table 1. A chest X-ray showed pulmonary interstitial edema/infiltrates (Figure 1). Peripheral blood smear showed 4% blasts and frequent dyspoietic granulocytes. Bone marrow biopsy (BMB) was performed out of concern for acute leukemia versus MDS.

**Table 1:** Initial laboratory values MCV: mean corpuscular volume; RDW: red blood cell distribution width; CRP: C-reactive protein; ESR: erythrocyte sedimentation rate

Laboratory tests	Value	Normal values
Hemoglobin - 2 weeks prior (g/dL)	7.3	13-17
Platelets - 2 weeks prior (x 10^3^/mL)	40	150-400
White blood cells (x 10^3^/mL)	9.83	4-10
Hemoglobin (g/dL)	5.7	13-17
Hematocrit (%)	17.2	40-52
Platelets (x 10^3^/mL)	27	150-400
MCV (fL/cell)	87.3	80-100
RDW (%)	22.6	11.5-14.5
Creatinine (mg/dL)	1.3	0.5-1.2
Ferritin (ng/mL)	2230	20-500
CRP (mg/L)	393.2	1-3
ESR (mm/hr)	>120	0-29

Following hospital admission, the patient was transfused both red blood cells and platelets and started on empiric antibiotic therapy with vancomycin and piperacillin-tazobactam. Labs covering a broad differential, including copper deficiency, hemophagocytic lymphohistiocytosis (HLH), acute leukemia, MDS, and septic shock secondary to pneumonia, were drawn. Lactate dehydrogenase (LDH) and haptoglobin were normal. Peripheral blood smear did not reveal evidence of hemolysis or hemophagocytosis. Copper was elevated at 196 µg/dL (reference range: 70-140 µg/dL). Fibrinogen was elevated to >900 mg/dL (reference range 200-400 mg/dL). HIV, hepatitis panel, coagulation panel, B12, and folate were within normal limits.

**Figure 2:**
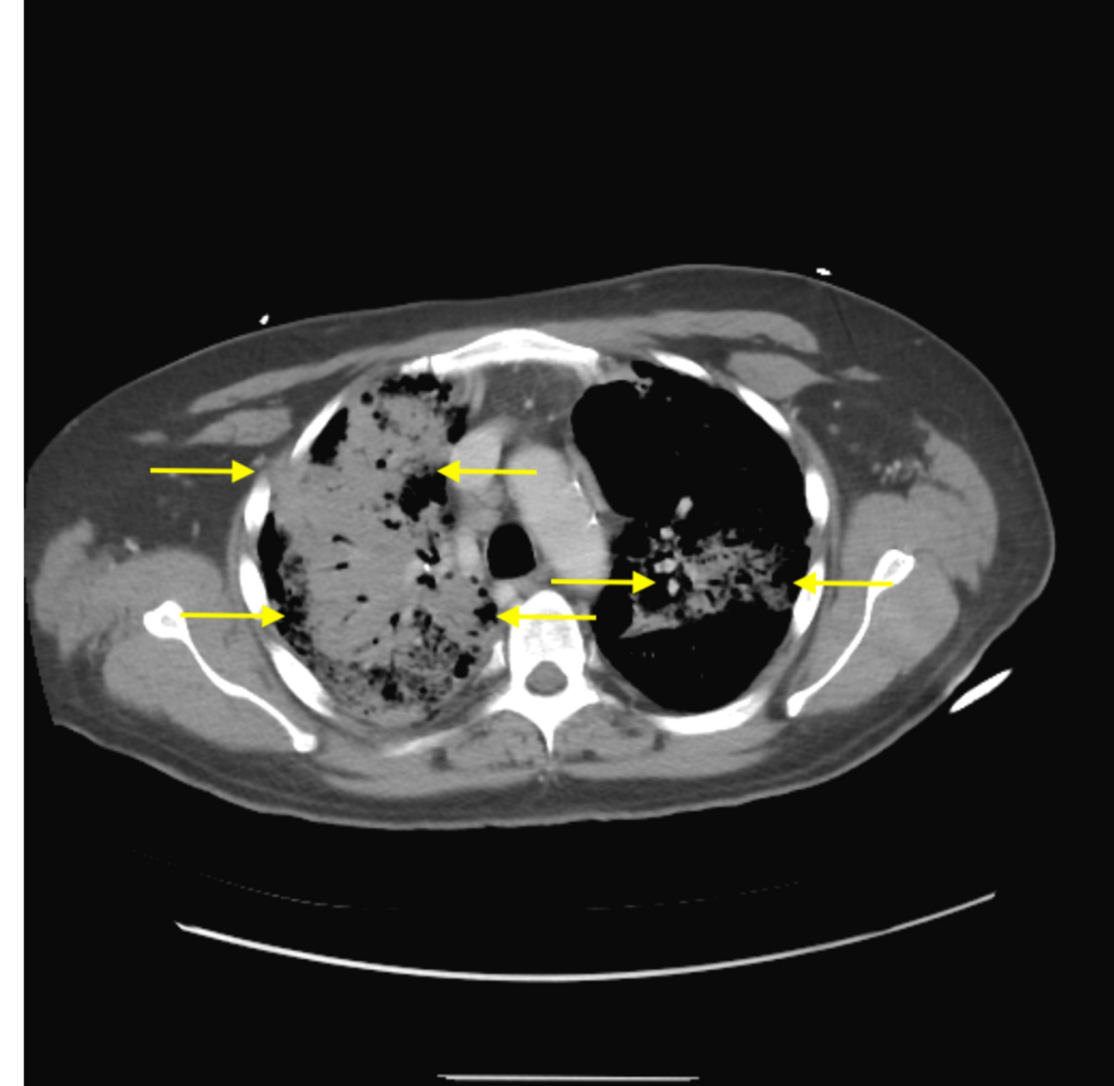
CT chest without contrast The image displays dense airspace consolidations (yellow arrows); BAL obtained shortly after radiography revealed diffuse alveolar hemorrhage CT: computed tomography; BAL: bronchoalveolar lavage

On hospital day five, the patient rapidly deteriorated and required transfer to the medical intensive care unit (ICU) for acute hypoxemic respiratory failure requiring intubation and mechanical ventilation. CT thorax showed airspace consolidations consistent with dense pneumonia (Figure 2). He underwent bronchoscopy with bronchoalveolar lavage (BAL), which showed 11,000 red blood cells but no microbial growth. He was diagnosed with diffuse alveolar hemorrhage secondary to thrombocytopenia, with a platelet count of 12 x 10^3^/mL. Because the patient was already on antibiotics at the time of BAL, he was escalated to meropenem, doxycycline, and amphotericin B out of concern for worsening sepsis. Antibiotics were continued until blood, urine, and BAL cultures returned negative on hospital day 11. With no improvement on antimicrobial therapy, sepsis as the underlying etiology was deemed less likely. However, the patient remained anemic and persistently thrombocytopenic despite multiple transfusions. With preliminary BMB results showing myelodysplasia, the differential shifted to an MDS-related process.

The patient was started on high-dose corticosteroids, equivalent to approximately 2 mg/kg prednisone, to treat a suspected paraneoplastic autoimmune vasculitis; this rare phenomenon has been described in the literature in patients with MDS [5]. The patient improved and was able to be extubated after four days. He no longer needed supplemental oxygen, required fewer transfusions, and was transferred out of the ICU. Labs showed a C-reactive protein (CRP) of 48 mg/L, platelet count of 58 x 10^3^/mL, and hemoglobin of 9 g/dL. CT chest revealed resolving airspace consolidation and post-inflammatory changes. An autoimmune panel showed a mildly elevated C3 of 172 mg/dL with normal C4. Antinuclear antibody (ANA) and rheumatoid factor were both negative.

**Figure 3:**
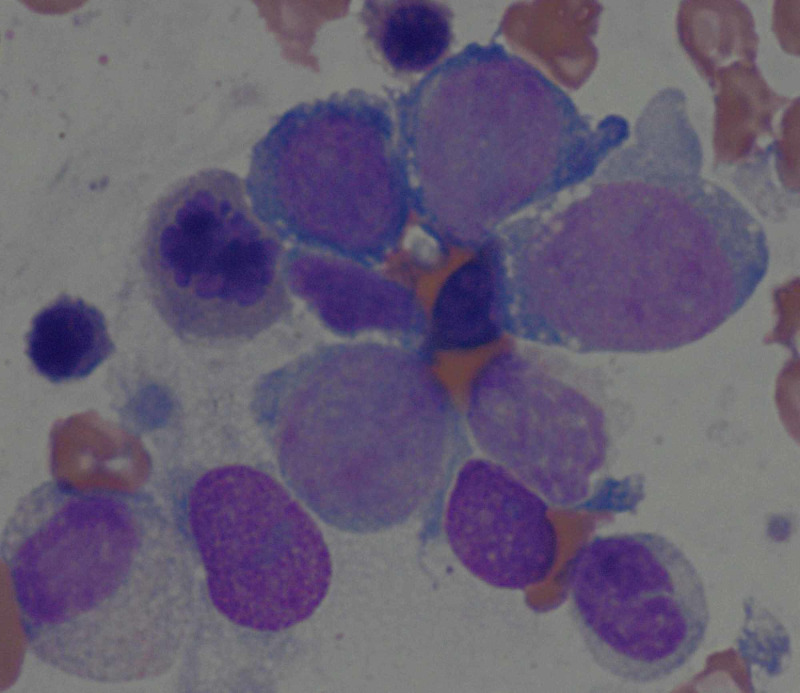
Bone marrow biopsy revealing myelodysplasia with excess blasts and erythroid predominance

The BMB revealed myelodysplasia with excess blasts and erythroid predominance (Figure 3). Blasts were quantified at 12%. Cytogenetic studies demonstrated a complex karyotype: 45,XY,add(5)(q22),-8,der(12)t(8;12)(q11.2;q15),-13,+mar{3}/43,idem, t(8;12)(p21;q15),del(16)(p13.1),-18,-20{13}/46,XY{13}. The patient was determined to be a candidate for chemotherapy that was not available on-site, so he was transferred to another facility. The patient had been hospitalized for 24 days at that point.

High-dose steroids were continued, and he was started on decitabine with the goal of bridging to bone marrow transplantation. On day three of decitabine therapy, dexamethasone was stopped. Within 48 hours, the patient developed cough and chest tightness with a chest X-ray suggestive of bilateral interstitial edema. On day five of decitabine, the patient developed scattered reticular opacities and nodular foci of consolidation as well as enlarged mediastinal lymph nodes on CT thorax. He became hypotensive with rising troponin levels, concerning for type II non-ST elevation acute coronary syndrome. The patient developed acute hypoxemic respiratory failure requiring intubation and mechanical ventilation. Diffuse alveolar hemorrhage was suspected, given his history, and high-dose steroids were restarted on ICU transfer. Hypotension persisted, warranting vasopressors. The patient experienced recurrent episodes of ventricular tachycardia requiring three separate episodes of cardiopulmonary resuscitation with defibrillation. At this point, the patient’s family decided to pursue comfort care; he was palliatively extubated and declared deceased one hour later. The family declined an autopsy. The patient had been hospitalized for a total of 36 days.

## Discussion

This case of paraneoplastic autoimmune vasculitis in a patient with MDS highlights diffuse alveolar hemorrhage as a rare but fatal complication. Autoimmune phenomena are demonstrable in up to 30% of MDS patients with varied manifestations [4]. In this patient, a broad differential initially included copper deficiency, HLH, acute leukemia, MDS, and septic shock secondary to pneumonia. 

Dysplasia on BMB remains the definitive method to diagnose MDS [5,6]. Key diagnostic criteria are dyspoietic morphologies involving more than 10% of one or more hematopoietic lineages, with or without increased blasts [6]. Because MDS has multiple mimickers and confounders, it is in some respects a diagnosis of exclusion. For example, deficiencies of copper, vitamin B12, and folate can present with anemia and leukopenia, and HIV has a wide variety of presentations [7]. Because this patient had elevated neutrophil counts, circulating blasts, and dyspoietic granulocytes but normal copper, B12, and folate levels, a true MDS or leukemia became more probable.

A diagnosis of HLH was considered in light of the patient’s fever, bicytopenia, and elevated ferritin. HLH is diagnosed if a patient meets five of the following eight criteria: temperature of ≥38.5°C, splenomegaly, cytopenias affecting two-thirds of cell lines (hemoglobin of <9 g/dL, platelets of <100 x 10^3^/mL, neutrophils of <1 x 10^3^/mL), fasting triglycerides of >265 mg/dL +/- fibrinogen of <150 mg/dL, hemophagocytosis, low or absent natural killer (NK)-cell activity, ferritin of >500 ng/mL, and elevated soluble CD25 (sCD25) [8]. Although the patient’s ferritin was greater than 2,000, levels over 3,000 are more suspicious, and levels over 10,000 are even more highly suspicious for HLH [8]. While the patient did have transient hypertriglyceridemia, this resolved with cessation of propofol. The patient’s fibrinogen was elevated to >900 mg/dL, the peripheral smear showed no features suggestive of hemolysis, and hemophagocytosis was not evident in the BMB. Including the mildly elevated ferritin, only three of eight HLH criteria were met; hence HLH was excluded as a possible diagnosis.

In the absence of an infectious source, the clinical constellation of polyarthralgia, edema, and pulmonary infiltrates in a patient with MDS is most probably due to paraneoplastic autoimmune vasculitis. A literature review revealed that the patient’s presentation was similar to that described by Enright et al. [9]. In that study involving 30 patients, common presentations of patients with MDS-associated autoimmune disorders included vasculitic rash (n = 15) and arthritis (n = 11), with seven of the 30 patients having a triad of vasculitic rash, fever, and arthritis. Of these seven patients, three had peripheral edema, and five had pulmonary infiltrates [9]. All but one patient responded well to immunosuppression with steroids, primarily prednisone [9]. Thus, this patient was promptly treated with high-dose steroids; a remarkable improvement in symptoms followed. The dramatic response to corticosteroids further reinforces the autoimmune nature of his pulmonary disease.

Killick et al. have reported that between 33 and 45% of high-risk MDS patients progress to acute myeloid leukemia (AML), having a “… median survival of around 12 months without intervention” [10]. Stem-cell transplantation is a mainstay of treatment, but transplant-ineligible patients may benefit from “… intensive AML-style chemotherapy …” [10] or hypomethylating agents [6,10]. Mekinian et al. have related that hypomethylating agents, such as azacytidine and decitabine, have not only shown promise in treating MDS effectively but have also demonstrated efficacy in treating cases of MDS-associated autoimmune disorders [11]. In our patient, decitabine was chosen as a bridge to definitive stem-cell transplantation. Because steroids are inherently immunosuppressive, the decision was made to stop steroids during treatment with decitabine. However, a major area for future study is the determination of the optimum duration of overlap between definitive therapy for MDS and steroid withdrawal. In this case, steroid withdrawal was perhaps performed too quickly, leading to the recurrence of fatal symptoms. Additional research is required to identify the ideal timing of various treatment modalities for patients with complications from an autoimmune paraneoplastic vasculitis secondary to MDS.

## Conclusions

This case highlights the fact that diffuse alveolar hemorrhage, the etiology of which is most likely a paraneoplastic autoimmune vasculitis, is a rare but potentially deadly complication of MDS. Providers caring for patients with MDS should be wary of such complications. In turn, MDS should be considered in patients who exhibit signs and symptoms suspicious for atypical autoimmune phenomena. Given the potential consequences, including death, early diagnosis in conjunction with specialist teams is imperative. Altogether, a paraneoplastic autoimmune vasculitis may be the underlying etiology of pulmonary complications in patients with MDS.
